# Gastric Composite Tumor of Alpha Fetoprotein-Producing Carcinoma/Hepatoid Adenocarcinoma and Endocrine Carcinoma with Reference to Cellular Phenotypes

**DOI:** 10.1155/2012/201375

**Published:** 2012-03-05

**Authors:** Akira Suzuki, Naohiko Koide, Masato Kitazawa, Akiyoshi Mochizuka, Hiroyoshi Ota, Shinichi Miyagawa

**Affiliations:** ^1^Department of Surgery, Shinshu University School of Medicine, 3-1-1 Asahi, Matsumoto 390-8621, Japan; ^2^Department of Clinical Laboratory Medicine, Shinshu University School of Medicine, 3-1-1 Asahi, Matsumoto 390-8621, Japan; ^3^Biomedical Laboratory Sciences, Shinshu University School of Medicine, 3-1-1 Asahi, Matsumoto 390-0802, Japan

## Abstract

Alpha-fetoprotein-producing carcinoma (AFPC)/hepatoid adenocarcinoma (HAC) and neuroendocrine carcinoma (NEC) are uncommon in the stomach. Composite tumors consisting of these carcinomas and their histologic phenotypes are not well known. Between 2002 and 2007, to estimate the prevalence of composite tumors consisting of tubular adenocarcinoma, AFPC/HAC and NEC, we reviewed specimens obtained from 294 consecutive patients treated surgically for gastric cancer. We examined histological phenotype of tumors of AFPC or NEC containing the composite tumor by evaluating immunohistochemical expressions of MUC2, MUC5AC, MUC6, CDX2, and SOX2. Immunohistochemically, AFPC/HAC dominantly showed the intestinal or mixed phenotype, and NEC frequently showed the gastric phenotype. In the composite tumor, the tubular and hepatoid components showed the gastric phenotype, and the neuroendocrine component showed the mixed type. The unique composite tumor predominantly showed the gastric phenotype, and the hepatoid and neuroendocrine components were considered to be differentiated from the tubular component.

## 1. Introduction

Alpha-fetoprotein (AFP) is a fetal protein produced by yolk sac cells, fetal hepatic cells, and some fetal gastrointestinal cells [1]. AFP-producing carcinoma (AFPC) is rare in the stomach [2], although gastric adenocarcinoma is one of the most common manifestations of AFPC [3]. Ishikura et al. [4] reported that hepatoid adenocarcinoma (HAC) is characterized by both hepatoid differentiation and AFP production, while the histological features of hepatoid differentiation in gastric AFPC have been determined. Gastric AFPC or HAC frequently displays aggressive behavior [2, 5]; however, these tumors exhibit many unresolved clinical and histopathological features. On the other hand, neuroendocrine carcinoma (NEC), including small cell carcinoma, is rare in the stomach [6], and its clinicopathological features and clinical outcome have been characterized recently [7, 8]. Both AFPC and NEC were classified as special types of gastric carcinomas by the Japanese Classification of Gastric Carcinoma [9]. Composite gastric tumors, consisting of special types of carcinoma, have been insufficiently investigated. A composite gastric tumor usually consists of both a common and a special type of carcinoma, that is, well- or moderately differentiated adenocarcinoma and HAC [4].

Gastric adenocarcinomas have been histopathologically classified into four categories based on their cellular phenotype [10]. Several cellular markers, including MUC2, MUC5AC, and MUC6, have been used to histochemically investigate these phenotypes. It has been identified that intestinal goblet cells, gastric foveolar epithelial cells, and pyloric gland cells cause the expression of MUC2 [11], MU5AC [12], and MUC6 [13], respectively. Measurement of CDX2 and SOX2 expression to investigate cellular phenotype has been adopted, recently. CDX2, homeobox gene has been known to play a role in the development of small and large intestines [14], while transcription factor SOX2 is expressed in normal stomach but not in the colon [15].

We examined the histological phenotypes of a composite gastric tumor consisting of AFPC/HAC, NEC, and tubular adenocarcinoma and investigated the cellular phenotypes of AFPC and NEC by evaluating the immunohistochemical expressions of MUC2, MUC5AC, MUC6, CDX2, and SOX2.

## 2. Material and Methods

### 2.1. Patients and Materials

To estimate the prevalence of composite gastric tumors consisting of tubular adenocarcinoma, AFPC/HAC, and NEC, we reviewed gastrectomy specimens obtained from 294 consecutive patients treated surgically for gastric cancer during a 5-year period (2002–2007) at Shinshu University Hospital. In addition to these consecutive case series, one case of composite gastric tumor of tubular adenocarcinoma, AFPC/HAC, and NEC was included. These specimens were examined after obtaining informed consent.

Three-micrometer-thick serial paraffin sections were obtained from representative formalin-fixed, paraffin-embedded blocks and stained with hematoxylin and eosin (HE) for histological examination or subsequent immunohistochemical staining to investigate their cellular phenotype.

### 2.2. Histopathological Diagnosis

Gastric AFPC was histopathologically diagnosed by a positive reaction of carcinoma cells in AFP immunostaining with or without hepatoid differentiation. NEC was suggested by the detection of undifferentiated carcinoma, including small cell carcinoma, by HE-staining, and diagnosed by a positive reaction of more than one-third of carcinoma cells for immunostaining with several neuroendocrine markers, including neural cell adhesion molecule (NCAM, CD56), chromogranin A (CGA), synaptophysin (SP), and neuron-specific enolase (NSE). Neuroendocrine markers were applied to tumors suspected of neuroendocrine phenotype based on examination of HE sections. According to the WHO 2010 classification of neuroendocrine neoplasms [16], NEC is diagnosed by immunostaining with Ki-67. Small cell carcinoma is defined as a tumor whose cells have scant cytoplasm, finely granular nuclear chromatin, measure less than the diameter of three small resting lymphocytes, and have faint nucleoli. Large cell carcinoma is defined as a tumor whose cells are large, with moderate to abundant cytoplasm and round to oval nuclei with frequent prominent nucleoli. All AFP and NEC cases were examined in three to six blocks. The clinicopathological features of gastric carcinomas are described in accordance with the WHO 2010 Classification of Tumours of the Stomach [16].

### 2.3. Immunohistochemistry and Classification of Cellular Phenotypes

The expression of MUC2, MUC5AC, MUC6, CDX2, and SOX2 was immunohistochemically investigated to confirm the presence cellular phenotypes of gastric AFPC or NEC. Microwave treatment was performed in 1 mM EDTA/10 mM Tris buffer (pH 8.0) for 25 min. The presence of more than one third of carcinoma cells in immunostained samples was defined as a positive reaction. The commercially available antibodies for this immunostaining are shown in Table 2. Carcinomas were classified into four categories based on their cellular phenotype in accordance with immunohistochemical findings; intestinal, mixed, gastric, or null (unclassified) phenotype [10]. This phenotypic classification, a modified version of the definition described by Kumashiro et al. [17], is summarized in Table 1.

## 3. Results

**Figure 1:**
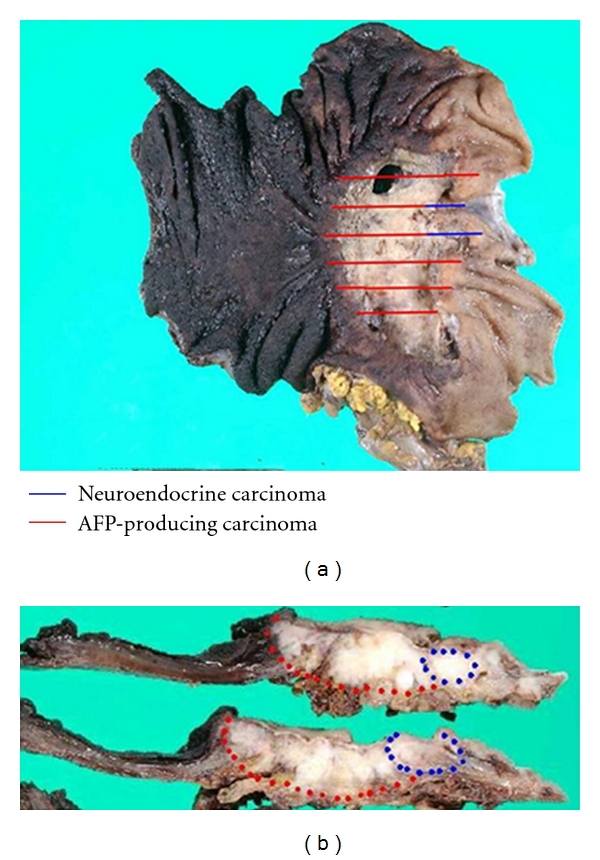
Macroscopic finding of the resected stomach in Case 1. A type 3 tumor, 75 × 110 mm in diameter, is located in the upper stomach. Gross photograph of cut sections is shown. Blue lines show a presence of NEC component, while red lines show a presence of the tubular and hepatoid components based on the histopathological findings.

**Figure 2:**
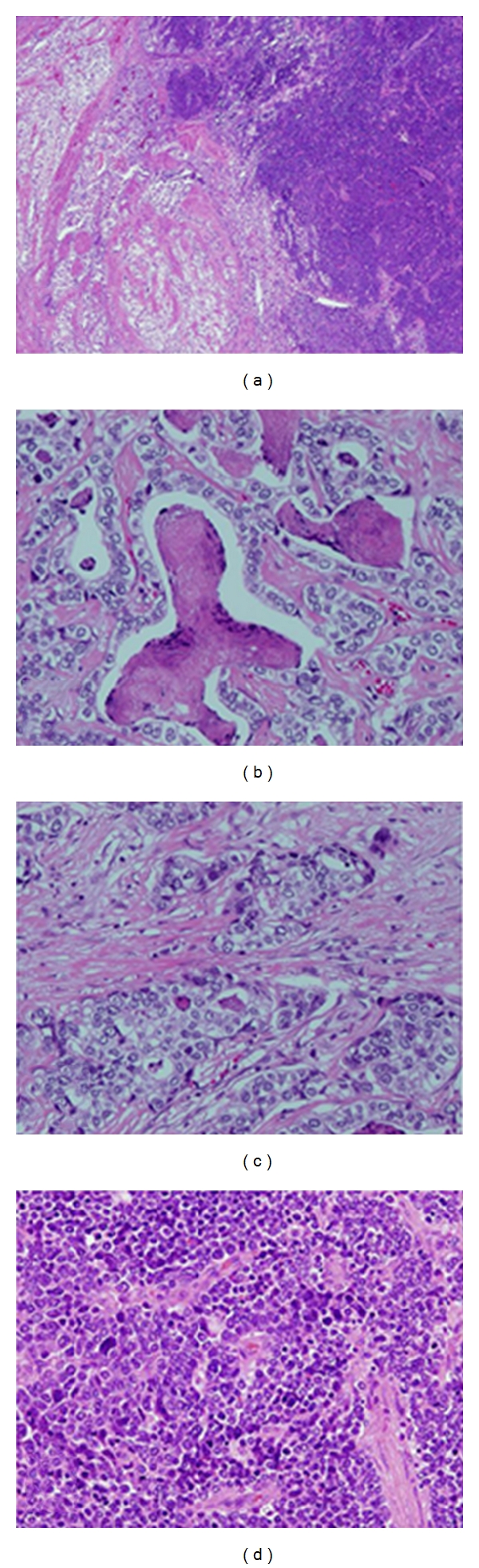
Histopathological findings of Case 1. Three types of carcinoma, including tubular adenocarcinoma, hepatoid adenocarcinoma, and neuroendocrine carcinoma, are shown. The left side of the photograph shows the tubular and hepatoid components, while the right side shows the neuroendocrine component. The tubular component is shown. The hepatoid component is shown. The neuroendocrine component is shown.

A composite gastric tumor consisting of AFPC/HAC, NEC, and tubular adenocarcinoma was observed in only one case (Case 1). In the resected specimen from this case, we observed an ulcerated tumor (75 × 110 mm) with no well-defined borders infiltrating into the surrounding tumor wall (Figure 1(a)). The NEC and AFPC components were located on the blue and red line, respectively, in cut sections of the resected specimen (Figure 1(b)). Histopathologically, this gastric carcinoma comprised of three types of components (Figure 2(a)). Moderately differentiated tubular adenocarcinoma was identified (tubular component; Figure 2(b)), while other type of carcinoma cells, with large eosinophilic and clear cytoplasm, had infiltrated with a trabecular or sheet-like pattern that displayed the morphological features of HAC (hepatoid component; Figure 2(c)). Furthermore, undifferentiated carcinoma cells had infiltrated the proximal part of the tumor. This component had polygonal carcinoma cells with prominent nucleoli in the nuclei, suggestive of large cell NEC (neuroendocrine component; Figure 2(d)). In this case, the NEC-cell component comprised of about 30% of the carcinoma, so this tumor was diagnosed not as neuroendocrine differentiation but as a gastric-mixed adenoneuroendocrine carcinoma (MANEC).

Clinicopathological features of patients with gastric AFPC/HAC or NEC, including the composite gastric tumor in Case 1, are shown in Table 3. Excluding Case 1, AFPC/HAC was observed in four cases (1.4%), while NEC was observed in six cases (2.0%). Among the six NEC cases, the large and small cell types of EC were observed in four and two cases, respectively. Among the four AFPC cases, HAC was observed in one case. There was no case of a composite gastric tumor consisting of AFPC/HAC and NEC, excluding Case 1. Synchronous expression of both AFP and neuroendocrine markers was not observed in any tumors; moreover, the cooccurrence of gastric AFPC and NEC was not observed in any tumors.

**Figure 3:**
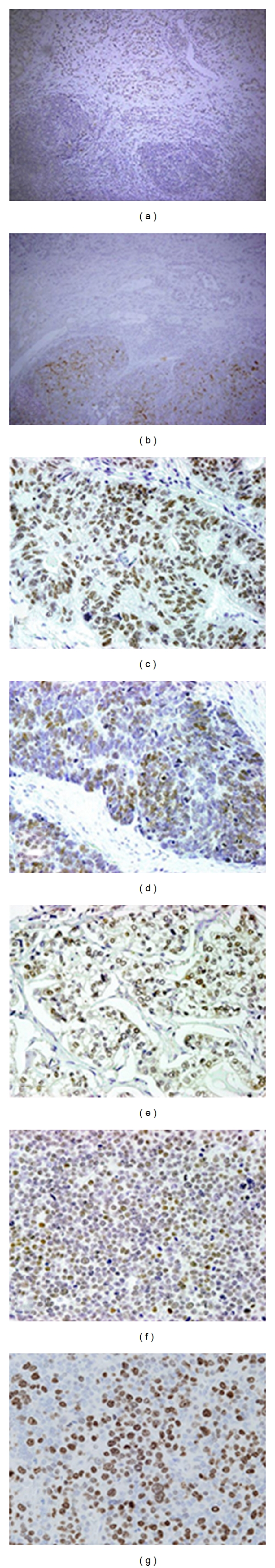
Immunohistochemistry for AFP-producing adenocarcinoma and neuroendocrine carcinoma. (a) AFP immunostaining in Case 1 (the tubular component of the composite tumor). A positive reaction for AFP is observed in the cytoplasm of the carcinoma cells. The upper side of the photograph shows the tubular and hepatoid components with a positive reaction for AFP, while the lower side shows the neuroendocrine component with a negative reaction for AFP. (b) Chromogranin A immunostaining in Case 1 (NEC component of the composite tumor). A positive reaction for synaptophysin is observed in the cytoplasm of the carcinoma cells. The upper side of the photograph shows the tubular and hepatoid components with a negative reaction for chromogranin A, while the lower side shows the neuroendocrine component with a positive reaction for chromogranin A. (c) CDX2 immunostaining in Case 8 (usual case of AFPC). A positive reaction for CDX2 is observed in the nuclei of tubular adenocarcinoma cells. (d) SOX2 immunostaining in Case 2 (usual case of NEC). A positive reaction for SOX2 is observed in the nuclei of neuroendocrine carcinoma cells. (e) SOX2 immunostaining in Case 1 (the tubular and hepatoid components). A positive reaction for SOX2 is observed in the nuclei of the carcinoma cells. (f) CDX2 immunostaining in Case 1 (NEC component). A positive reaction for CDX2 is observed in the nuclei of the carcinoma cells. (g) Ki-67 immunostaining in Case 1 (NEC component). More than 20% carcinoma cells are positive for Ki-67.

Immunohistochemical findings for diagnosis of composite gastric tumor, AFPC/HAC and NEC, are shown in Table 4. All six NEC cases manifested a positive reaction for one or more neuroendocrine markers, while no reaction was evidenced for AFP. SP was expressed in all NEC cases, while NCAM, CGA, and NSE were expressed in three NEC cases. These neuroendocrine markers were completely expressed in only one NEC case. None of the four AFPC/HAC cases evidenced a reaction with neuroendocrine markers. In Case 1, the tubular and hepatoid components evidenced a positive reaction for AFP (Figure 3(a)), while no reaction was observed for neuroendocrine markers. The neuroendocrine component of composite gastric tumor demonstrated positive reactions for CGA (Figure 3(b)) and SP, while no reaction was observed for AFP.

Immunohistochemical findings for the cellular phenotypes of composite gastric tumor, AFPC/HAC, and NEC are shown in Table 4. MUC2 was expressed in one AFPC case, while MUC5AC and MUC6 were expressed in the other three cases. CDX2 was expressed in all AFPC/HAC cases (Figure 3(c)), while SOX2 was expressed in one AFPC case. MUC2 was expressed in one NEC case, MUC5AC was expressed in the other NEC case, and MUC6 in four NEC cases. CDX2 was not expressed in any of the NEC cases, while SOX2 was expressed in five NEC cases (Figure 3(d)). In NEC cases, MUC6 was expressed in almost the same site when compared to neuroendocrine markers. MUC2 and MUC5AC were expressed in the part of the positive site associated with the neuroendocrine markers, but in different areas to each other. In Case 1, the tubular component evidenced no reaction for MUC2, MUC5AC, or MUC6, but expressed SOX2 (Figure 3(e)), and the hepatoid component expressed MUC6 and SOX2. The neuroendocrine component did not express MUC2, but expressed MUC5AC, MUC6, SOX2, and CDX2 (Figure 3(f)). In all NEC cases, more than 20% carcinoma cells are positive for Ki-67 (Figure 3(g)).

Consequently, with reference to the cellular phenotypes in EC, gastric, mixed, and null phenotypes were observed in four, one, and one case, respectively. In AFPC/HAC, the intestinal and mixed phenotypes were observed in cases one and three, respectively. In the composite gastric tumor, the tubular and hepatoid components evidenced the gastric phenotype, while the neuroendocrine component showed the mixed phenotype.

## 4. Discussion

The composition of gastric AFPC/HAC and NEC is not yet understood clinicopathologically. Only a few cases [18–20] of gastric carcinoma consisting of AFPC/HAC and NEC have been reported. Okamaoto et al. [18] reported a composite gastric tumor consisting of AFPC and NEC, similar to the present case. Rassidakis et al. [19] reported gastric HAC with extensive neuroendocrine differentiation, and Ueda et al. [20] reported a composite tumor consisting of poorly differentiated adenocarcinoma and NEC with synchronous AFP expression. In the present study, no case of composite gastric tumor consisting of AFPC/HAC and NEC was observed, excluding Case 1. Furthermore, no case of gastric carcinoma with synchronous expression of AFP and neuroendocrine markers was observed. It was considered that this composition may be sporadic, and its occurrence may be very rare.

 Gastric HAC frequently contains well- or moderately differentiated adenocarcinoma including tubular adenocarcinoma [4, 21, 22]. Kishimoto et al. [23] suggested that HAC developed from tubular adenocarcinoma, and Akiyama et al. [21] reported that gastric HAC and adenocarcinomatous components are of monoclonal origin. In the present study, although HAC concomitant with adenocarcinoma was observed in only one case, AFP was expressed in well- or moderately differentiated adenocarcinomas. On the other hand, mixed glandular exocrine tumors or composite neuroendocrine exocrine tumors have been identified in the stomach [24, 25], and NEC has frequently been associated with an adenocarcinoma component in the stomach [24–26]. These tumors have been defined as adenocarcinoma and NEC in combination in the same tumor. NEC predominantly arises from endocrine precursor cell clones occurring in preceding adenocarcinoma components [26]. In other tumors, such as pancreatic carcinomas [27], HAC was found to be associated with another cellular component, either NEC or ductal carcinoma. Furthermore, several cases of hepatocellular carcinoma with neuroendocrine differentiation have been reported [28]. In the present study, six gastric NEC cases were of the histologically pure type with no glandular component. Although, as mentioned above, cases such as the composite case presented are rare, gastric adenocarcinoma may also have the potential to differentiate into other miscellaneous types of carcinoma including either AFPC or NEC, such as in this case.

Based on immunohistochemistry results for cellular phenotype, Kumashiro et al. [17] recently reported that HAC and adenocarcinomatous components mainly evidenced the intestinal phenotype, and that the gastric phenotype was not observed in HAC. In the present study, AFPC/HAC expressed both intestinal and mixed phenotypes. On the other hand, the cellular phenotype has not yet been analyzed sufficiently in gastric NEC. Iwafuchi et al. [29] reported that, in Japanese subjects, gastric NEC frequently showed the intestinal phenotype in immunohistochemical analyses for CD10, MUC2, MUC5AC, and MUC6, with the complete intestinal phenotype constituting 45.5% of cases and incomplete intestinal phenotype consisting of 6.1% of the cases in gastric NEC. In the present study, four of the six EC cases evidenced the gastric phenotype immunohistochemically. In the composite tumor, both the hepatoid and the tubular components demonstrated the gastric phenotype immunohistochemically. The neuroendocrine component showed the presence of the mixed phenotype immunohistochemically, but this finding was considered as a gastric dominant type because MUC5AC, MUC6, and SOX2 were expressed in the neuroendocrine component. The finding that NEC evidenced the gastric dominant phenotype could be useful as a reference. These findings for cellular phenotype suggest that the hepatoid and neuroendocrine components may differ from the tubular component.

SOX2 has been shown to be downregulated in intestinal metaplasia in the stomach [30], and its expression was demonstrated in gastric adenocarcinoma with both gastric and mixed phenotype [31]. CDX2 has been shown to be expressed in intestinal development [14] and was demonstrated in intestinal metaplasia and adenocarcinoma with the intestinal phenotype of the stomach [32, 33]. In the present study, gastric NEC frequently expressed SOX2 but not CDX2, while gastric AFPC/HAC frequently expressed CDX2 but not SOX2. These NEC cases showed the presence of the gastric phenotype, but AFPC/HAC may be considered as being strongly associated with the intestinal phenotype. In the composite tumor, the three components expressed SOX2, but CDX2 was expressed in the neuroendocrine component. Therefore, this composite gastric tumor was considered to be unique and different from typical gastric NEC or AFPC/HAC cases.

In conclusion, this unique composite gastric tumor predominantly evidenced the gastric phenotype, and the hepatoid and neuroendocrine components were considered to be differentiated from the tubular component. Generally, gastric AFPC/HAC predominantly evidenced the intestinal phenotype or mixed type, and NEC frequently showed the gastric phenotype.

## Figures and Tables

**Table 1:** Phenotypic classification. Gastric carcinomas were classified four categories as follows; intestinal, mixed, gastric, and null (unclassified) phenotypes. These categories were decided by immunohistochemical finding of MUC2, MUC5AC, and MUC6, and CDX2 and SOX2.

	MUC5AC(−), and MUC6(−), and SOX2(−)	MUC5AC(+), or MUC6(+), or SOX2(+)
MUC2(+) or CDX2(+)	Intestinal type	Mixed type
MUC2(−) and CDX2(−)	Null type	Gastric type

**Table 2:** Antibodies used for immunohistochemistry.

Antigen	Clone	Source	Dilution	Preparation
Neural cell adhesion molecule	123C3 (mm)	Dako	×50	Microwave
Chromogranin A	A0430 (rp)	Dako	×200	—
Synaptophysin	A0010 (rp)	Dako	×100	Microwave
Neuron-specific enolase	BBS/NC/VI-H14 (mm)	Dako	×1 (kit)	Microwave
Alfa-Fetoprotein	A0008 (rp)	Dako	×300	Microwave
Ki-67	MIB-1 (mp)	Dako	×100	Microwave
MUC2	CCP58 (rp)	Novocastra	×200	Microwave
MUC5AC	45M1 (rp)	Novocastra	×300	Microwave
MUC6	CLH5 (rp)	Novocastra	×200	Microwave
CDX2	CDX2-88 (mm)	BioGene	×300	Microwave
SOX2	GT15098 (rp)	Neuromi CS	×1000	Microwave

mm, mouse monoclonal antibody; rp, rabbit polyclonal antibody.

**Table 3:** Clinicopathologic features of AFP-producing carcinoma and neuroendocrine carcinoma of the stomach.

Case	Diagnosis	Age	Sex	Site	Size (mm)	Histology	T	N	M	Outcome (months)
1	MANEC	83	M	U	75 × 110	Tub 2 > hepatoid > large cell	4	2	1	DOD (6)
2	LCNEC	81	M	M	25 × 20	Large cell	2	0	0	DOD (12)
3	LCNEC	74	M	U	50 × 50	Large cell	2	3	0	DOD (13)
4	LCNEC	77	M	U	90 × 80	Large cell	3	1	0	DOD (8)
5	LCNEC	62	M	L	45 × 30	Large cell	2	1	0	alive (30)
6	SCNEC	54	F	U	9 × 8	Small cell	1	0	0	alive (66)
7	SCNEC	63	M	U	65 × 60	Small cell	3	2	1	alive (24)
8	AFPC	69	M	U	40 × 32	Pap > tub 1	1	0	0	alive (30)
9	AFPC	69	M	U	60 × 50	Por > hepatoid	3	1	0	alive (42)
10	AFPC	70	M	L	19 × 17	Tub 1 > tub 2	1	0	0	alive (66)
11	AFPC	51	M	M	35 × 14	Tub 2 > tub 1	1	0	1	alive (24)

MANEC, mixed-adenoneuroendocrine carcinoma;

LCNEC, large-cell neuroendocrine carcinoma;

SCNEC, small-cell neuroendocrine carcinoma;

AFPC, alfa fetoprotein-producing carcinoma;

Site; U, upper-third; M, middle-third; L, lower-third of the stomach.

Tub 1, well-differentiated tubular adenocarcinoma;

Tub 2, moderately differentiated tubular adenocarcinoma;

Pap, papillary adenocarcinoma;

Por, poorly differentiated adenocarcinoma;

DOD, died of disease.

**Table 4:** Immunohistochemical findings of AFP-producing carcinoma and neuroendocrine carcinoma of the stomach.

Case	Histology	NCAM	CGA	SP	NSE	AFP	MUC2	MUC5AC	MUC6	CDX2	SOX2	Ki67 labeling index (%)	Phenotype
	Tubular	−	−	−	−	+	−	−	−	−	+	16	Gastric
1	Hepatoid	−	−	−	−	+	−	−	+	−	+	12	Gastric
	LCNEC	−	+	+	−	−	−	+	+	+	+	42	Mixed
2	LCNEC	+	+	+	−	−	−	−	+	−	+	24	Gastric
3	LCNEC	+	+	+	+	−	−	−	+	−	+	32	Gastric
4	LCNEC	+	−	+	−	−	+	+	+	−	+	22	Mixed
5	LCNEC	−	−	+	−	−	−	−	−	−	+	23	Gastric
6	SCNEC	−	−	+	+	−	−	−	−	−	−	32	Null
7	SCNEC	−	+	+	+	−	−	−	+	−	+	25	Gastric
8	AFPC	−	−	−	−	+	+	−	−	+	−	35	Intestinal
9	AFPC	−	−	−	−	+	−	+	+	+	−	18	Mixed
10	AFPC	−	−	−	−	+	−	+	+	+	+	8	Mixed
11	AFPC	−	−	−	−	+	−	+	+	+	−	12	Mixed

LCNEC, large-cell neuroendocrine carcinoma;

SCNEC, small-cell neuroendocrine carcinoma;

AFPC, alfa fetoprotein-producing carcinoma;

NCAM, neural-cell adhesion molecule;

CGA, chromogranin A;

SP, synaptophysin;

NSE, neuron-specific enolase;

AFP, alfa fetoprotein.
